# Use of a Handheld Ultrasonographic Device to Identify Heart Failure and Pulmonary Disease in Rural Africa

**DOI:** 10.1001/jamanetworkopen.2024.0577

**Published:** 2024-02-28

**Authors:** Andrew Katende, Johanna Oehri, Victor Z. Urio, Evance Mahundi, Lulu Wilson, Victor Myovela, Chipegwa Mlula, Christamonica Chitimbwa, Caspar Mbawala, Fanuel Faustine, Valentine Mteki, Winfrid Gingo, Faraja Kitila, Ipyana Mwasongwe, Claudia Bucher, Luigia Elzi, James Okuma, Thomas Zoller, Daniel H. Paris, Maja Weisser, Martin Rohacek

**Affiliations:** 1 St Francis Regional Referral Hospital, Ifakara, United Republic of Tanzania; 2 Ifakara Health Institute, Ifakara, United Republic of Tanzania; 3 Swiss Tropical and Public Health Institute, Allschwil, Switzerland; 4 University of Basel, Basel, Switzerland; 5 Regional Hospital of Bellinzona e Valli, Bellinzona, Switzerland; 6 Department of Infectious Diseases and Respiratory Medicine, Charité–Universitätsmedizin Berlin, corporate member of Freie Universität Berlin and Humboldt-Universität zu Berlin, Berlin, Germany; 7 Division of Infectious Diseases and Hospital Epidemiology, University Hospital Basel, Basel, Switzerland

## Abstract

**Importance:**

Agreement in lung ultrasonography findings between clinicians using a handheld ultrasonographic device and expert sonographers using a high-end ultrasonographic machine has not been studied in sub-Saharan Africa.

**Objective:**

To determine the agreement in ultrasonographic findings and diagnoses between primary care clinicians trained in lung ultrasonography, board-certified expert sonographers, and senior physicians.

**Design, Setting, and Participants:**

This cross-sectional single-center study was conducted from February 1, 2022, to April 30, 2023 at a referral center in rural Tanzania. Individuals 5 years or older with respiratory symptoms and at least 2 distinct respiratory signs or symptoms were eligible. A total of 459 individuals were screened.

**Exposures:**

Participants provided their medical history and underwent a clinical examination and lung ultrasonography performed by a clinician, followed by a lung ultrasonography performed by an expert sonographer, and finally chest radiography and a final evaluation performed by a senior physician. Other tests, such as echocardiography and *Mycobacterium tuberculosis* testing, were conducted on the decision of the physician. Clinicians received 2 hours of instruction and three 2-hour sessions of clinical training in the use of a handheld lung ultrasonographic device; expert sonographers were board-certified.

**Main Outcomes and Measures:**

Percentage agreement and Cohen κ coefficient for sonographic findings and diagnoses compared between clinicians and expert sonographers, and between clinicians and senior physicians.

**Results:**

The median (IQR) age of 438 included participants was 54 (38-66) years, and 225 (51%) were male. The median (range) percentage agreement of ultrasonographic findings between clinicians and expert sonographers was 93% (71%-99%), with κ ranging from −0.003 to 0.83. Median (range) agreement of diagnoses between clinicians and expert sonographers was 90% (50%-99%), with κ ranging from −0.002 to 0.76. Between clinicians and senior physicians, median (range) agreement of diagnoses was 89% (55%-90%), with κ ranging from −0.008 to 0.76. Between clinicians and senior physicians, diagnosis agreements were 85% (κ, 0.69) for heart failure, 78% (κ, 0.57) for definite or probable tuberculosis, 50% (κ, 0.002) for viral pneumonia, and 56% (κ, 0.06) for bacterial pneumonia.

**Conclusions and Relevance:**

In this cross-sectional study, the agreement of ultrasonographic findings between clinicians and sonographers was mostly substantial. Between clinicians and senior physicians, agreement was substantial in the diagnosis of heart failure, moderate in the diagnosis of tuberculosis, but slight in the diagnosis of pneumonia. These findings suggest that handheld ultrasonographic devices used in addition to clinical examination may support clinicians in diagnosing cardiac and pulmonary diseases in rural sub-Saharan Africa.

## Key Points

**Question:**

Can trained primary care clinicians in rural Africa use handheld ultrasonographic devices to accurately diagnose cardiopulmonary conditions?

**Findings:**

In this cross-sectional study including 438 patients, there was mostly substantial agreement of ultrasonographic findings between clinicians using a handheld ultrasonographic device and expert sonographers using a high-end ultrasonographic machine. Between clinicians and senior physicians, there was a substantial agreement in the diagnosis of heart failure, moderate agreement in the diagnosis of tuberculosis, but only slight agreement in the diagnosis of pneumonia.

**Meaning:**

These findings suggest that handheld ultrasonographic devices may support clinicians in rural Africa in diagnosing pulmonary and cardiac diseases.

## Teaser

This cross-sectional study assesses the use of handheld lung ultrasonographic devices by trained primary care clinicians to diagnose cardiopulmonary conditions in rural Africa.

## Introduction

Lung ultrasonography, a point-of-care ultrasonography (POCUS) examination of the chest, has gained importance as a bedside diagnostic tool, especially during the COVID-19-pandemic.^1,2,3^ The use of POCUS is important especially in resource limited settings, where other imaging technologies, such as conventional radiology and computed tomography, are not readily available. The technique can be learned by a dedicated training scheme in a time-efficient way^4^ and can support clinicians in performing patient triage and diagnosis in rural settings.^5^ The combination of lung and heart ultrasonography in patients with acute dyspnea may provide a higher diagnostic accuracy than chest radiography in differentiating cardiac vs pulmonary disease.^6^ Lung ultrasonography has been shown to offer a good sensitivity and specificity to diagnose pneumonia (94% and 96%),^7^ pleural effusion (78%-89% and 88%-99%) and congestive heart failure (96% and 88%) if used by adequately trained individuals.^8^ During the COVID-19-pandemic, lung ultrasonography was shown to be a useful tool for triage and quick diagnostic workup.^1,2,3^ As technology is improving with portable and affordable ultrasonography devices with high image quality,^9^ lung ultrasonography may become an important diagnostic tool for clinicians, in addition to history taking and physical examination.

In sub-Saharan Africa, sonography of the chest has mainly focused on tuberculosis, with specified protocols to detect signs of extrapulmonary tuberculosis.^10,11^ In children, several studies have shown the added value of sonography for tuberculosis and other pulmonary diseases.^12,13,14^ At an urban emergency department in Dar es Salaam, Tanzania, respiratory symptoms were the second most frequent reason for sonography after trauma. The use of chest sonography enabled identification of pathological findings in 54 of 64 patients (84%) and affected treatment management for 45% of patients.^5^

The use of handheld ultrasonographic devices accelerates the diagnostic work-up.^15^ The introduction of portable devices in an African emergency department^16^ and in a refugee camp in Tanzania^17^ showed advantages, such as low cost and maintenance. Even though these studies involved relatively large sample sizes,^5,17^ they lacked quality control imaging,^5,16^ and no outcome data of the patients were reported.^5,16,17^ There are knowledge gaps on which patient population may benefit most from the use of handheld ultrasonographic devices^18^ and what training protocols are needed to establish a clinically useful lung ultrasonography training curriculum.^17^ To date, it is not known if clinicians who receive a short training in lung ultrasonography with a handheld ultrasonography device reach the same diagnoses of pulmonary and cardiac diseases compared with experts in sonography using a high-end ultrasonographic machine.

We hypothesized that trained clinicians using a handheld ultrasonographic device in addition to clinical examination would be able to detect pathological sonographic signs comparable to an expert sonographer, and to make diagnoses of diseases of the chest comparable to a senior physician who determined the final diagnoses.

## Methods

### Study Design and Setting

This cross-sectional single-center study was conducted from February 1, 2022, to April 30, 2023, at the St Francis Regional Referral Hospital (SFRRH), Ifakara, in rural Tanzania. The aim was to determine the agreement of sonographic findings and diagnoses between clinicians performing lung ultrasonography with a handheld ultrasonographic device and expert sonographers using a high-end ultrasonographic machine. Moreover, we determined the agreement of diagnoses between clinicians, expert sonographers, and senior physicians. Ethical clearance for the study was obtained from the institutional ethical review board of the Ifakara Health Institute and from the National Institute of Medical Research of Tanzania. All patients provided written informed consent after receiving the study information in the Swahili language. For participants aged 5 to 18 years, or participants unable to consent due to illness, an additional consent was sought from the legal guardian. If no legal guardian was available, the patient was not included. This study followed the Strengthening the Reporting of Observational Studies in Epidemiology (STROBE) reporting guideline for cross-sectional studies.

The SFRRH is the referral center for about 1 million people living in the Kilombero, Ulanga, and Malinyi districts of the Kilombero valley and offers inpatient care for 370 patients. It runs specialized services in emergency medicine, internal medicine, surgery, obstetrics and gynecology, pediatrics, neonatology, and ophthalmology and has an HIV and tuberculosis clinic. Since May 2021, SFRRH has included a specialized clinic for heart and lung diseases. Besides routine services, SFRRH has established a training program for POCUS and echocardiography.^19^

### Training in Use of the Device

Preceding the study start, clinicians who were not experienced in sonography received 2 hours of theoretical instruction and 3 sessions of a 2-hour practical training on how to perform lung ultrasonography using the handheld ultrasonographic device. Additionally, e-learning material (videos) on lung ultrasonography were distributed for self-study. The training was conducted by board-certified sonographers and was completed by taking a practical exam to assess the understanding of the taught lung ultrasonography principles. Lung ultrasonography was performed according to a standard operating procedure, with sonography of the inferior vena cava (IVC) and a subcostal view of the heart by convex probe added to a previously published protocol.^20^ Moreover, the lung was not only examined with the linear probe (or lung preset of the handheld device), but also with the convex probe (or abdominal pre-set of the handheld device). This was performed in 6 thoracic positions (anterior, lateral, and posterior on both sides) in long- and short-axis views. Trainees were trained to detect dilated noncollapsing IVC or a depressed left ventricular function and 3 or more B-lines bilaterally as a sign for heart failure; a larger right than left ventricular diameter as a sign for right heart failure^21^; pericardial effusion; pleural effusion in supine and sitting positions; B-lines as a nonspecific sign for an interstitial pathological finding, such as interstitial edema or interstitial pneumonia; hypoechogenic consolidations with air trapping as a sign for infiltrates^22^; and hepatization as a sign for lobar or bacterial pneumonia; B-lines in combination with bright subpleural granular artifacts as a sign for tuberculosis^23^; thick B-lines in combination with subpleural consolidations and fragmentation of the pleura together with a collapsible IVC as a sign for interstitial or viral pneumonia^24^; and pneumothorax, defined as absence of pleural sliding, absence of lung pulse, presence of lung point, or presence of barcode sign.^25^

Devices used in this study were 2 handheld ultrasonographic devices (Butterfly iQ+; Butterfly Network, Inc) connected to a tablet; the devices are licensed in Europe and the US. The expert sonographer used standard of care ultrasonographic machines (Mindray M7, Chison Sonobook 9).

### Study Participants and Recruitment

Adults and children 5 years or older attending any of the departments of the SFRRH were eligible for study participation if they had at least 2 signs or symptoms of respiratory illness (ie, dyspnea, dry or productive cough of any duration, chest pain, oxygen saturation <92%, or crackles on auscultation). Exclusion criteria were refusal to participate, no written informed consent, younger than 5 years, or severe acute disease requiring immediate medical intervention. Clinicians referred all patients with respiratory symptoms to the study team for further evaluation for study inclusion after initial treatment and stabilization.

### Study Procedures

Two senior physicians assessed the eligibility of the participants and initiated the consent process. Patients who provided informed consent offered a full medical history and then underwent a physical examination and lung ultrasonography with a handheld ultrasonographic device conducted by a nonradiologist clinician who was a medical doctor, clinical officer (ie, a health care worker who joined a nonuniversity medical school for 3 years), or nurse. Thereafter, an experienced sonographer (ie, expert sonographer) who received information about the patient’s history and physical examination repeated lung ultrasonography using a high-end ultrasonographic machine. All sonographic findings and differential diagnoses were documented on a prepared hard copy of a case report form. More than 1 diagnosis could be selected when several differential diagnoses were possible. After lung ultrasonography, participants underwent chest radiography. A comprehensive echocardiogram conducted by experienced echocardiographers according to current recommendations^26^ was performed if deemed necessary by the senior physician. The senior physician interviewed and physically examined the participant in a separate independent session and made a final diagnosis based on their own judgment, medical information, and results of the lung ultrasonography from both examiners. The clinician and the expert sonographer were blinded from the results of each other and did not have access to the results of other investigations, such as chest radiograph, echocardiogram, or *Mycobacterium tuberculosis* testing (using Xpert MTB/RIF Ultra; Cepheid), until after they had documented their findings. The senior physician determined the final diagnosis and decided the treatment of the patient per standard guidelines. In cases with discrepancies between the 2 lung ultrasonography approaches that could lead to changes in management, the physician was allowed to repeat the lung ultrasonography. In total, there were 9 trained clinicians (4 medical doctors [J. Oehri, C. Mlula, C.C., and I.M.], 3 clinical officers [E.M., C. Mbawala, and F.F.], and 2 nurses [V. Mteki and C.B.]), 4 expert sonographers, including (V. Myovela, F.K., M.R.) performing lung ultrasonography, and 3 senior physicians (A.K., V.Z.U, and M.R.). Medical doctors had 1 to 4 years’ experience, clinical officers 2 to 3 years’ experience, and nurses 2 to 10 years’ clinical experience. Experienced sonographers were board-certified in POCUS and had 4 to 14 years of experience. Senior physicians were board-certified in internal medicine or pediatrics and had clinical experience of 8 to 23 years. Detailed information about qualification and experience of medical personnel involved is available in the eAppendix in Supplement 1.

### Sample Size Calculation

Assuming that a difference of 10% or lower in the performance of a diagnosis based on full sonography vs POCUS is clinically irrelevant, and that full sonography has 90% probability of correct diagnosis,^10,27^ the calculated sample size was 438 patients with 80% power and an α of 5%. The sample size calculation was performed using Stata, version 14 for Windows (StataCorp LLC).

Based on data from the emergency department treating 35 903 patients in 1 year (2016-2017) indicating that 12.6% presented with respiratory tract infection,^28^ the enrollment was estimated to last 1 year. Of note, the number of patients visiting the emergency department increased to 88 000 in 2021.^29^

### Data Management

All data were transferred from physical case report forms to an electronic database (EpiData) by a data manager (E.M.). To ensure utmost accuracy and minimize errors, a second data manager conducted a thorough review after the data were entered into the electronic database.

### Statistical Analysis

Medians, ranges, and IQRs were used to describe continuous data, and frequencies and proportions were used to describe categorical data. The outcomes of the percentage agreement and interrater reliability for lung ultrasonographic findings and diagnoses between clinicians, sonographers, and physicians were calculated. Cohen κ statistic was interpreted as follows: <0, no agreement; 0 to 0.20, slight agreement; 0.21 to 0.40, fair agreement; 0.41 to 0.60, moderate agreement; 0.61 to 0.80, substantial agreement; 0.81 to 1, almost perfect agreement. Sensitivity, specificity, positive predictive value, and negative predictive value were calculated for lung ultrasonography findings of clinicians vs expert sonographers. All tests were 2-tailed. Data analysis was performed using Stata, version 16 (StataCorp LLC).

## Results

A total of 459 patients were screened, of which 438 were enrolled and analyzed; 10 patients could not be enrolled because they were in serious condition, 9 patients because they did not fulfill inclusion criteria, and 2 patients refused to participate. The median (IQR) age was 54 (38-66) years, 225 (51%) were male, 213 (49%) were female, 55 (13%) tested HIV positive, and 185 (42%) were inpatients. A total of 104 participants (24%) had a documented history of a cardiac disease, and 79 participants (18%) had a history of tuberculosis. The most frequent symptom reported was dyspnea (345 [79%]) followed by dry cough in 226 participants (52%). The predominant clinical findings included elevated blood pressure in 295 participants (67%) and pulmonary crackles in 329 participants (75%) (Table 1).

**Table 1.** Baseline Characteristics of 438 Study Participants

Characteristic	Participants, No. (%)
Sociodemographics	
Age, median (IQR), y	54 (38-66)
Age category, y	
<18	20 (5)
18-54	208 (47)
≥55	210 (48)
Sex	
Male	225 (51)
Female	213 (49)
Pregnancy (in 213 females)	4 (2)
Occupation	
Farmer	330 (80)
Other^a^	85 (20)
Missing^b^	23 (5)
HIV status	
Negative	383 (87)
Positive	55 (13)
Care type	
Inpatient	185 (42)
Outpatient	253 (58)
Medical history	
Asthma	27 (6)
COPD	5 (1)
Cardiac disease	104 (24)
Active tuberculosis	31 (7)
Receiving antituberculosis medication	30 (97)
History of tuberculosis	79 (18)
Presenting symptom	
Dyspnea	345 (79)
Cough^c^	
Dry	226 (52)
Productive	172 (39)
Chest pain	267 (61)
Fever	145 (33)
Weight loss	242 (55)
Weight gain	59 (13)
Palpitations	136 (31)
Clinical finding	
BMI	
Median (IQR)	21.9 (19.3-25.4)
Category	
Underweight (<18.5)	87 (20)
Normal weight (18.5 to <25)	231 (53)
Overweight (25 to <30)	74 (17)
Obese (≥30)	46 (11)
Blood pressure	
Normal	143 (33)
Elevated (ie, ≥140/90 mm Hg)	295 (67)
Pulmonary signs	
Oxygen saturation <92%	68 (16)
Oxygen saturation, median (IQR)	97 (95-98)
Pulmonary crackles	329 (75)
Wheezing	40 (9)
Dullness of percussion	82 (19)
Diminished breath sound	93 (21)
Heart murmur	133 (30)
Arrhythmia^d^	71 (16)
Cardiac signs	
Heart rate, median (IQR), beats/min	95 (82-107)
Distended jugular veins	35 (8)
Lower limb edema	123 (28)

Abbreviations: BMI, body mass index (calculated as weight in kilograms divided by height in meters squared); COPD, chronic obstructive pulmonary disease.

^a^ Including 4 miners (1%).

^b^ Results are No. (%) of participants with non-missing data; missing data are No. (column percentage).

^c^ Cough includes cough of any duration.

^d^ Arrythmia includes irregular heartbeat or tachycardia.

A total of 101 participants underwent lung ultrasonography conducted by a medical doctor, 219 by a clinical officer, and 118 by a nurse; 2 of the 4 expert sonographers performed lung ultrasonography only 5 times (eTable 1 in Supplement 1). Table 2 shows the diagnoses made by clinicians, expert sonographers, and senior physicians. The most common final diagnoses determined by the physician were bacterial pneumonia in 240 participants (55%), heart failure in 180 participants (41%), and probable or definite tuberculosis in 175 participants (40%), including 57 participants with post-tuberculosis sequelae. eTable 2 in Supplement 1 shows additional examinations exclusively available to the physician, including 408 participants (93%) who underwent chest radiography and 286 participants (65%) who underwent a comprehensive echocardiogram, for which findings from 216 participants (76%) were outside normal ranges (eTable 3 in Supplement 1). In cases in which tuberculosis was suspected, Xpert MTB/RIF Ultra was performed for 126 participants. Tests using Xpert MTB/RIF Ultra were conducted for 92 participants (21%) with sputum, 37 (8%) with pleural fluid, and 10 (2%) with urine.

**Table 2.** Diagnoses Stratified by Clinician, Expert Sonographer, and Physician Findings for 438 Participants

Diagnosis	Participants, No. (%)^a^		
	Clinicians^b^	Expert sonographers^c^	Physicians^d^
Heart failure	181 (41)	172 (39)	180 (41)
Tuberculosis	220 (50)	153 (35)	175 (40)^e^
Miliary	4 (1)	9 (2)	10 (2)
Pulmonary	160 (37)	132 (30)	132 (30)
Pleural	87 (20)	15 (3)	83 (19)
Extra pulmonary	93 (21)	27 (6)	92 (21)
Post sequelae	72 (16)	56 (13)	57 (13)
Pneumonia			
Bacterial	346 (79)	380 (87)	240 (55)
Viral	218 (50)	397 (91)	88 (20)
COPD			
With cor pulmonale	34 (8)	27 (6)	31 (7)
Without cor pulmonale	13 (3)	6 (1)	23 (5)
Pleural effusion			
With empyema	15 (3)	18 (4)	16 (4)
Without empyema	179 (41)	183 (42)	175 (40)
Lung cancer	1 (0.2)	1 (0.2)	18 (4)
Pericardial effusion	76 (17)	48 (11)	58 (13)
Pneumothorax	7 (2)	5 (1)	5 (1)
Chronic interstitial lung disease	102 (23)	51 (12)	52 (12)
Pulmonary embolism	5 (1)	9 (2)	11 (3)
Metastatic disease	2 (0.5)	5 (1)	11 (3)
Other heart disease	85 (19)	74 (17)	132 (30)

Abbreviation: COPD, chronic obstructive pulmonary disease.

^a^ Total diagnoses may exceed 100% because more than 1 diagnosis per participant was possible.

^b^ Results from clinicians trained in using a handheld ultrasonographic device include clinical and handheld ultrasonographic device findings.

^c^ Results from experienced board-certified sonographers include clinical and regular lung ultrasonography findings.

^d^ Results from physicians include clinical, lung ultrasonography, and chest radiography findings.

^e^ Xpert MTB/RIF Ultra conducted in 126 participants, with negative results in 100 and positive results in 26.

In total, 26 of 118 (22%) participants with probable or definite (ie, microbiologically confirmed) active tuberculosis had a positive result from at least 1 of the tested sites. Of these 26 participants, clinicians detected definite tuberculosis in 25 participants (96%).

Median (range) agreement of ultrasonographic findings between clinicians and expert sonographers was 93% (71%-99%), with κ ranging from −0.003 to 0.83. There was almost perfect agreement (92%; κ, 0.83; sensitivity, 88%; and specificity 95%) to detect pleural effusion dorsal right, substantial agreement (83%; κ, 0.65; sensitivity, 82%; and specificity 84%) to detect normal heart function, substantial agreement (85%; κ, 0.63; sensitivity, 95%; and specificity, 64%) to detect a collapsing IVC, moderate agreement (71%; κ, 0.42; sensitivity, 77%; and specificity, 64%) to detect infiltrates, and fair agreement (73%; κ, 0.33; sensitivity, 65%; and specificity, 75%) to detect subpleural granular artifacts (Table 3). For the detection of seashore sign on the left side (agreement, 98%; κ, 0.3) and lung pulse (agreement, 99%; κ, −0.004), the percentage agreement was high despite the low κ. However, there were 5 participants with pneumothorax only. Median (range) agreement of diagnoses between clinician and expert sonographer was 90% (50%-99%), with κ ranging from −0.002 to 0.76; between clinician and physician, the median (range) agreement of diagnoses was 89% (55%-90%), with κ ranging from −0.008 to 0.76 (Table 4). Between clinician and senior physician, agreement for diagnoses of heart failure was 85% (κ, 0.69), definite or probable tuberculosis was 78% (κ, 0.57), pneumonia (not specified) was 60% (κ, 0.06), viral pneumonia was 50% (κ, 0.002), and bacterial pneumonia was 56% (κ, 0.06) (Table 5). Between expert sonographer and physician, median (range) agreement of diagnoses was 92% (29%-99%), with κ ranging from −0.004 to 0.83. Between expert sonographer and physician, the agreement for diagnoses of heart failure was 92% (κ, 0.83), definite or probable tuberculosis was 83% (κ, 0.63), pneumonia (not specified) was 61% (κ, 0.05), viral pneumonia was 29% (κ, 0.04), and bacterial pneumonia was 58% (κ, 0.11) (eTable 4 in Supplement 1).

**Table 3.** Comparison of Lung Ultrasonography Findings Between Clinicians and Expert Sonographers for 438 Participants

Lung ultrasonography finding	Clinicians, No. (%)	Expert sonographers, No. (%)	Sensitivity, %	Specificity, %	PPV, %	NPV, %	Agreement, %	Cohen κ
Inferior vena cava collapsing	334 (76)	301 (69)	95	64	85	85	85	0.63
Pericardial effusion	80 (18)	50 (11)	82	89	51	97	88	0.55
Normal heart function	194 (44)	177 (40)	82	84	89	77	83	0.65
Pleural effusion								
Lateral left	154 (35)	150 (34)	85	91	83	92	89	0.76
Lateral right	150 (34)	156 (36)	87	95	90	92	92	0.81
Dorsal left	151 (34)	150 (34)	84	91	83	92	89	0.75
Dorsal right	149 (34)	154 (35)	88	95	91	93	92	0.83
Fibrin strands^a^	68 (15)	54 (12)	67	92	53	95	89	0.52
Internal echoes^b^	19 (4)	19 (4)	32	97	32	97	94	0.28
B-lines	430 (98)	424 (97)	99	21	97	38	96	0.26
Infiltrates	256 (58)	238 (54)	77	64	72	70	71	0.42
Air trapping^c^	52 (12)	58 (13)	33	92	37	90	84	0.25
Hepatization of lung tissue	14 (3)	19 (4)	53	99	71	98	97	0.59
Mass	1 (0.2)	3 (0.7)	NA	NA	NA	NA	99	−0.003
Subpleural granular artifacts	146 (33)	94 (21)	65	75	42	89	73	0.33
Fragmentation of pleura	102 (23)	99 (23)	52	85	50	86	77	0.36
Pleura thickening	67 (15)	22 (5)	41	86	13	97	84	0.14
Lung sliding								
Anterior right	431 (98)	434 (99)	99	50	99	29	98	0.36
Anterior left	432 (99)	432 (99)	99	33	99	40	98	0.32
Seashore								
Anterior right	431 (98)	434 (99)	99	75	99	43	99	0.54
Anterior left	432 (99)	429 (98)	99	25	99	40	98	0.3
Barcode								
Anterior right	7 (2)	4 (1)	75	99	43	99	99	0.54
Anterior left	5 (1)	6 (1)	NA	NA	NA	99	98	0.36
Lung pulse	437 (100)	433 (99)	99	0	99	0	99	−0.004
Lung point	5 (1)	3 (0.7)	NA	NA	NA	NA	99	0.24
Rib fracture	1 (0.2)	0	NA	NA	NA	NA	NA	NA

Abbreviations: NA, not applicable because of low numbers; NPV, negative predictive value; PPV, positive predictive value.

^a^ Structure attached to the lung or pleura floating in pleural fluid.

^b^ Hyperechogenic dots in pleural fluid suggesting high cell content (ie, empyema).

^c^ Hyperechogenicities in a consolidation suggesting air bronchogram.

**Table 4.** Comparison of Diagnoses Between Clinicians and Expert Sonographers for 438 Participants

Diagnosis	Clinicians, No. (%)	Expert sonographers, No. (%)	Agreement, %	Cohen κ
Tuberculosis	220 (50)	153 (35)	78	0.57
Miliary	4 (1)	9 (2)	97	−0.01
Pulmonary	160 (37)	132 (30)	78	0.50
Extrapulmonary	93 (21)	27 (6)	80	0.19
Post sequelae	72 (16)	56 (13)	94	0.76
Pneumonia	371 (85)	410 (94)	80	−0.03
Bacterial	346 (79)	380 (87)	74	0.09
Viral	218 (50)	397 (91)	50	0.01
Pulmonary embolism	5 (1)	9 (2)	98	0.28
COPD				
With cor pulmonale	34 (8)	27 (6)	92	0.38
Without cor pulmonale	13 (3)	6 (1)	96	0.09
Chronic interstitial lung disease	102 (23)	51 (12)	77	0.22
Pneumothorax	7 (2)	5 (1)	99	0.49
Lung or pleural cancer	1 (0.2)	1 (0.2)	99	−0.002
Metastatic disease	2 (0.5)	5 (1)	98	−0.007
Heart failure	181 (41)	172 (39)	86	0.71
Other heart disease	85 (19)	74 (17)	73	0.10

Abbreviation: COPD, chronic obstructive pulmonary disease.

**Table 5.** Comparison of Diagnoses Between Clinicians and Senior Physicians for 438 Participants

Diagnosis	Clinicians, No. (%)	Senior physicians, No. (%)	Agreement, %	Cohen κ
Tuberculosis	220 (50)	175 (40)	78	0.57
Miliary	4 (1)	10 (2)	97	−0.01
Pulmonary	160 (37)	132 (30)	76	0.46
Extrapulmonary	93 (21)	92 (21)	81	0.43
Post sequelae	72 (16)	57 (13)	93	0.74
Pneumonia	371 (85)	262 (60)	60	0.06
Bacterial	346 (79)	240 (54)	56	0.06
Viral	218 (50)	88 (20)	50	0.002
Pulmonary embolism	5 (1)	11 (3)	98	0.49
COPD				
With cor pulmonale	34 (8)	31 (7)	89	0.28
Without cor pulmonale	13 (3)	23 (5)	92	0.42
Chronic interstitial lung disease	102 (23)	52 (12)	76	0.18
Pneumothorax	7 (2)	5 (1)	99	0.49
Lung or pleural cancer	1 (0.2)	18 (4)	96	−0.004
Metastatic disease	2 (0.5)	11 (3)	97	−0.008
Heart failure	181 (41)	180 (41)	85	0.69
Other heart disease	85 (19)	132 (30)	67	0.14

Abbreviation: COPD, chronic obstructive pulmonary disease.

In addition, all analyses were conducted for medical doctors, clinical officers, and nurses separately. The results were broadly similar for all 3 groups.

## Discussion

The results of this cross-sectional study conducted in rural Tanzania suggest that handheld ultrasonographic devices used by clinicians trained in lung ultrasonography, in addition to history and physical examination, may be useful to detect ultrasonographic findings and to find the cause for a respiratory problem. Agreement in sonographic findings between clinicians and expert sonographers were mostly substantial, except for the interpretation of infiltrates and pleural abnormalities such as subpleural granular artifacts, in which agreements were moderate to fair. While there was substantial agreement between clinicians and senior physicians in the diagnosis of heart failure, agreement was moderate in the diagnosis of tuberculosis, and only slight in the diagnosis of pneumonia, especially in the differentiation between the bacterial or viral origin of pneumonia.

The successful training of clinicians in lung ultrasonography is in line with other reports. In a training program in remote Sierra Leone, clinicians were trained successfully in POCUS of the chest and abdomen and in extended focused assessment with sonography in trauma (eFAST).^30^ Training in POCUS for assessing pediatric respiratory diseases was successfully conducted in a center in South Sudan.^31^ In a study from Tanzania, clinicians were trained in POCUS of the heart during a 5-day training program.^32^ Nonexperts in echocardiography were able to screen for rheumatic heart disease using a handheld echocardiography device in Sudan,^33^ and POCUS findings with a handheld device were found to be reliable in detecting urgent obstetric conditions.^34^ In all of these reports, the generated images were deemed appropriate by the expert reviewers of the images, with substantial agreement. However, in those studies, except the last study mentioned,^34^ ultrasonographic images were reviewed remotely by experts who did not scan patients themselves. To the best of our knowledge, our study is the first to compare the lung ultrasonography performance of clinicians using handheld ultrasonographic devices with expert sonographers using a high-end ultrasonographic machine. This novelty adds evidence that handheld ultrasonographic devices used by nonexperts can be used in rural sub-Saharan African settings to diagnose patients with respiratory concerns. In particular, these findings support triage decisions made by clinicians examining patients first in a rural or remote setting, for example, to make a decision whether to refer the patient or to treat locally based on lung ultrasonography findings.

To describe the agreement of lung ultrasonography findings and diagnoses, we used percentage agreement and Cohen κ. While percentage agreement can be understood as the proportion of data that are correct, the κ value represents the amount of agreement that can be expected to be from random chance.^35^

Therefore, percentage agreement and κvalues may show different levels of agreement, as in the rating of the seashore sign and lung pulse in this study, for example. These findings are easy to detect in the absence of pneumothorax, and were detected correctly in almost all patients by both clinicians and sonographers, because participants with pneumothorax were rare. This resulted in a very high percentage agreement, but in a very low κ. Both percentage agreement and κ were high for the assessment of the IVC, pleural effusion, and heart function and for the diagnosis of heart failure. On the other hand, both kind of agreements were lower for the assessment of infiltrates and abnormalities of the pleura, and for the diagnosis of pneumonia.

This limitation of ultrasonography is of importance especially for clinicians working alone in rural remote areas. While the diagnosis of heart failure justifies a straightforward initiation of heart failure medication, a diagnosis of pneumonia may require additional tests, such as *M tuberculosis* testing of sputum, chest radiography, and consultation with a physician, for example, by telemedicine.

### Limitations

Our study has limitations. First, the final diagnoses performed by the physician depended on the findings and tests conducted on the day of recruitment, and follow-up of participants was not available. Thus, we could have missed some participants with heart failure, especially those with heart failure with preserved ejection fraction. Most of the diagnoses of tuberculosis were based on clinical and radiologic findings, while only 26 of 118 active cases were microbiologically confirmed. In addition, the lack of bacteriologic and virologic pathogen identification likely reduced the reliability of clinical diagnoses, such as tuberculosis and bacterial or viral pneumonia.

Second, we did not use a validated lung ultrasonography protocol, such as the BLUE protocol, because this protocol was developed in a resource-rich setting for critically ill patients.^27^ However, we used similar lung ultrasonography findings, such as B-lines, pleural effusion, consolidations, and signs for pneumothorax as used in the BLUE protocol. Third, individuals who performed lung ultrasonography were not blinded to patient history and physical examination findings, and physicians were not blinded to lung ultrasonography findings. However, this was a decision to represent everyday practice of clinicians and physicians using ultrasonography in addition to clinical findings and their judgment. Fourth, we could not determine if the differences between clinicians and expert sonographers were due to lung ultrasonographic skills or to technical differences between the handheld ultrasonographic device and high-end ultrasonographic machine. Fifth, included were symptomatic participants experiencing a respiratory disorder, and almost half were hospitalized. Therefore, generalizability to a less ill population is limited.

## Conclusions

In conclusion, this cross-sectional study showed substantial agreement for most pathological lung ultrasonography findings between experienced sonographers and clinicians who received a 2-hour instruction and three 2-hour sessions of clinical training in the use of a handheld ultrasonographic device. Between clinicians, expert sonographers and senior physicians, there was substantial agreement of diagnoses of heart failure, moderate agreement of diagnoses of probable or microbiologically confirmed tuberculosis, but only slight agreement of diagnoses of pneumonia. These findings suggest that handheld ultrasonographic devices used in addition to clinical examination may support clinicians in diagnosing cardiac and pulmonary diseases in rural sub-Saharan Africa.

## Notes

Supplement 1. **eAppendix.** Qualification and experience of medical personnel involved in the study **eTable 1.** LUS done by four medical doctors, three clinical officers, and two nurses **eTable 2.** Diagnostic tests done in 438 participants **eTable 3.** Findings of 286 comprehensive echocardiograms **eTable 4.** Comparison of diagnoses between expert sonographer and senior physician in 438 participants

Supplement 2.
Data Sharing Statement
