# Supplementary material for: Use of a Handheld Ultrasonographic Device to Identify Heart Failure and Pulmonary Disease in Rural Africa
Source: JAMA Netw Open. 2024 Feb 28;7(2):e240577. doi: 10.1001/jamanetworkopen.2024.0577 (PMC10902720; doi:10.1001/jamanetworkopen.2024.0577)
Supplement: Supplement 1. — eAppendix. Qualification and experience of medical personnel involved in the study eTable 1. LUS done by four medical doctors, three clinical officers, and two nurses eTable 2. Diagnostic tests done in 438 participants eTable 3. Findings of 286 comprehensive echocardiograms eTable 4. Comparison of diagnoses between expert sonographer and senior physician in 438 participants [file jamanetwopen-e240577-s001.pdf]

Supplementary Online Content

Katende A, Oehri J, Urio VZ, et al. Use of a handheld ultrasonographic device to identify heart failure and pulmonary disease in rural Africa. *JAMA Netw Open*. 2024;7(2):e240577. doi:10.1001/jamanetworkopen.2024.0577

eAppendix. Qualification and experience of medical personnel involved in the study
eTable 1. LUS done by four medical doctors, three clinical officers, and two nurses
eTable 2. Diagnostic tests done in 438 participants
eTable 3. Findings of 286 comprehensive echocardiograms
eTable 4. Comparison of diagnoses between expert sonographer and senior physician in 438 participants

This supplementary material has been provided by the authors to give readers additional information about their work.

for all normal and for JN-OA use: © 2024 American Medical Association. All rights reserved.
For CC-BY license, CC-BY-NC-ND open access, or JNO article use this: © 2024 last name first initials et al. *JAMA Network Open*.

eAppendix. Qualification and experience of medical personnel involved in the study

Clinicians

Medical doctors had successfully finished their studies in human medicine at a Tanzanian university lasting six years, and had one, three, three, and four years of clinical experience, respectively; clinical officers had successfully finished a non-university medical school (i.e three years at the Tanzanian Training Center for International Health (TTCIH, https://www.ttcih.ac.tz/), and had two, two, and three years of clinical experience, respectively. Nurses had successfully finished a nursing school, and had clinical experience of two and ten years, respectively. One medical doctor who performed four LUS only during the study did a course in POCUS before start of the study, all other clinicians did not have any experience in ultrasound before the start of the study.

Expert sonographers

Experienced sonographers were board certified in POCUS. Two had four years, one 8 years and one 14 years of experience in performing ultrasound.

Sonographer 1 (Victor Myovela) is an assistant radiologist and certified sonographer. He finished his 3-year training in ultrasound at the Kilimanjaro Christian Medical Centre (KCMC) Radiology Department in 2017. In addition, he was trained by Martin Rohacek since 2016 in point of care sonography and echocardiography. In daily practice, he does comprehensive echocardiograms according to current international guidelines.

Sonographer 2 (Martin Rohacek) is performing ultrasound and echocardiograms in daily practice since 2009. He is a certified instructor in POCUS and abdominal ultrasound by SGUM (Swiss Society for Ultrasound), and is a member of EFSUMB (European Federation of Societies for Ultrasound in Medicine and Biology). Since 2018, he is an organizer of regular courses in POCUS (EFSUMB endorsed), echocardiography, and vascular ultrasound in Ifakara since 2018.

Sonographer 3 and 4 are clinical officers working at the Emergency Department of the St Francis Regional Referral Hospital. They are certified in POCUS by EFSUMB, and have four years of experience in sonography.

Physicians

Physician 1 (Martin Rohacek) is a board-certified specialist in Internal Medicine and Emergency Medicine. He has 23 years of experience in clinical Internal Medicine.

Physician 2 (Victor Z. Urio) has a master in pediatrics from Makerere University, Kampala, Uganda, and worked for two years at the heart and lung clinic in Ifakara mostly with adult patients. During that time, he performed echocardiograms and LUS during daily practice in adults and children.

Physician 3 (Andrew Katende) has a master in internal Medicine from Makerere University, Kampala, Uganda, and has an experience of 8 years in clinical Internal Medicine. Since May 2021, he performs regular echocardiograms according to international guidelines and point of care ultrasound in daily practice. He is the head of the heart and lung clinic in Ifakara.

All who perform echocardiograms were trained by Martin Rohacek and by Cardiologists from the Department of Cardiology, University Hospital Basel, Basel, Switzerland.

eTable 1. LUS done by four medical doctors, three clinical officers, and two nurses

	Clinician, N=	Expert Sonographer N=	Physician, N=
MD1	51	N/A	N/A
MD2	29	N/A	N/A
MD3	17	N/A	N/A
MD4	4	N/A	N/A
CO1	24	N/A	N/A
CO2	70	N/A	N/A
CO3	125	N/A	N/A
Nurse1	113	N/A	N/A
Nurse1	5	N/A	N/A
Sonographer 1	N/A	400	N/A
Sonographer 2/Physician 1*	N/A	33	10
Sonographer 3	N/A	4	N/A
Sonographer 4	N/A	1	N/A
Physician 2	N/A	N/A	211
Physician 3	N/A	N/A	217

MD, Medical Doctor; CO, clinical Officer. * Role of either physician or expert sonographer

eTable 2. Diagnostic tests done in 438 participants

	Total	abnormal/positive	normal/negative
Chest X ray (n, %)	408	338 (83%)	70 (17%)
Echocardiography (n, %)*	286	215 (75%)*	71 (25%)
Xpert MTB/RIF Ultra Sputum (n, %)	92	22 (25%)	70 (75%)
Xpert MTB/RIF Ultra Pleura (n, %)	37	5 (14%)	32 (86%)
Xpert MTB/RIF Ultra Urine (n, %)	10	3 (30%)	7 (70%)

*140 had impaired left ventricular systolic function

eTable 3. Findings of 286 comprehensive echocardiograms

	N=
Total echocardiograms	286
Severe HF (LVEF<30%)	52
Moderate HF (LVEF 30-40%)	72
Mild HF (LVEF 41-50%)	17
Diastolic relaxation impairment grade 2 or 3	120
Diastolic relaxation impairment grade 1	21
Concentric remodeling	17
Eccentric left ventricular hypertrophy	90
Concentric left ventricular hypertrophy	33
Moderate or severe MR	66
Hypertensive heart disease	46
Valvular heart disease	22
Coronary heart disease	3
Other heart disease	53
Cor pulmonale/pulmonary hypertension*	37
Pericardial effusion	35

A total of 216 echocardiograms were abnormal. Diastolic relaxation impairment grade 2 or 3 indicate elevated left atrial pressure. * pulmonary hypertension, not caused by elevated left atrial pressure. HF, heart failure; LVEF, left ventricular ejection fraction; MR, mitral regurgitation.

eTable 4. Comparison of diagnoses between expert sonographer and senior physician in 438 participants

**Diagnosis**	**Expert Sonographer N (%)**	**Senior Physician N (%)**	**Agreement (%)**	**Kappa**
Tuberculosis	153 (35)	175 (40)	83	0.63
- Miliary tuberculosis	9 (2)	10 (2)	97	0.30
- Pulmonary tuberculosis	132 (30)	132 (30)	80	0.52
- Extrapulmonary tuberculosis	27 (6)	92 (21)	81	0.23
Post- tuberculosis sequelae	56 (13)	57 (13)	92	0.66
Pneumonia	410 (94)	262 (60)	61	0.05
- Bacterial	380 (79)	240 (54)	58	0.11
- Viral	397 (91)	88 (20)	29	0.04
Pulmonary Embolism	9 (2)	11 (3)	98	0.49
COPD				
- With cor pulmonale	27 (6)	31 (7)	96	0.67
- Without cor pulmonale	6 (1)	23 (5)	94	0.11
Chronic interstitial lung disease	51 (12)	52 (12)	88	0.42
Pneumothorax	5 (1)	5 (1)	99	0.60
Lung- or pleural cancer	1 (0.2)	18 (4)	96	-0.004
Metastatic disease	5 (1)	11 (3)	98	0.37
Heart failure	172 (39)	180 (41)	92	0.83
Other heart disease	74 (17)	132 (30)	73	0.26

Agreement, percentage agreement; kappa, Cohen's kappa; COPD, chronic obstructive pulmonary disease.
