# Supplementary material for: Use of a Handheld Ultrasonographic Device to Identify Heart Failure and Pulmonary Disease in Rural Africa
Source: JAMA Netw Open. 2024 Feb 28;7(2):e240577. doi: 10.1001/jamanetworkopen.2024.0577 (PMC10902720; doi:10.1001/jamanetworkopen.2024.0577)
Supplement: Supplement 2. — Data Sharing Statement [file jamanetwopen-e240577-s002.pdf]

Data Sharing Statement

Katende. Use of a Handheld Ultrasonographic Device to Identify Heart Failure and Pulmonary Disease in Rural Africa. *JAMA Netw Open*. Published February 28, 2024. doi:10.1001/jamanetworkopen.2024.0577

Data
Data available: Yes
Data types: Deidentified participant data
How to access data: Data will be available on request at mrohacek@ihi.or.tz
When available: With publication

Supporting Documents
Document types: Informed consent form
How to access documents: Documents will be available on request at mrohacek@ihi.or.tz
When available: With publication

Additional Information
Who can access the data: Data will be available to anyone upon request
Types of analyses: Metaanalysis
Mechanisms of data availability: Data will be available with investigator support
Any additional restrictions: N/A
